# Platelet function testing in pigs using the Multiplate® Analyzer

**DOI:** 10.1371/journal.pone.0222010

**Published:** 2019-08-29

**Authors:** Sarah Heringer, Lisa Kabelitz, Martin Kramer, Omid Nikoubashman, Marc A. Brockmann, Stefanie Kirschner, Martin Wiesmann

**Affiliations:** 1 Department of Diagnostic and Interventional Neuroradiology, RWTH Aachen University Hospital, Aachen, Germany; 2 Department of Veterinary Clinical Sciences, Small Animal Clinic, Justus-Liebig-University, Giessen, Germany; 3 Department of Neuroradiology, University Medical Center of the Johannes Gutenberg University Mainz, Germany; Instituto Mexicano del Seguro Social (IMSS) HGZ 2, MEXICO

## Abstract

For endovascular research pigs are an established animal model. However, experiences regarding analyses of platelet inhibition in pigs using the Multiplate® Analyzer are limited. The aims of the present study were to investigate if (1) the Multiplate® Analyzer is a suitable method for examination of porcine platelet function using manufacturers’ recommendations for human blood, and (2) platelet inhibition can be induced with acetylsalicylic acid (ASA) and clopidogrel in pigs reliably, and if (3) non-responders to one of the drug can be detected. Additionally we examined differences in (4) the effectiveness of ASA between oral administration and intravenous application, and (5) between domestic pigs (German Landrace; GL) and miniature pigs (MP). We investigated platelet function of 36 unmedicated pigs (GL n = 28; MP n = 8). In addition, 32 blood samples taken from medicated pigs (GL n = 15; MP n = 17) were analysed. Platelet inhibition was induced in four different ways: (1) 500 mg ASA intravenously (n = 11), (2) 500 mg ASA intravenously and 450 mg clopidogrel orally (n = 5), (3) 250 mg ASA orally (n = 11), (4) 250 mg ASA orally and 75 mg clopidogrel orally (n = 5). Results of the ASPI and ADP test of the Multiplate® Analyzer subtests in unmedicated and medicated pigs were in a comparable range to results known from humans. Application of ASA decreased the mean values of the ASPI test significantly regardless of the application method. Joined administration of ASA and clopidogrel also decreased the mean values of the ADP test significantly. Both, oral and intravenous administrations of ASA as well as oral administration of clopidogrel effectively inhibited platelet function in pigs. One pig did not respond to clopidogrel. We found no differences between domestic and miniature pigs regarding reference values in unmedicated pigs and the effectiveness of ASA and clopidogrel.

## Introduction

In humans, dual platelet inhibition with acetylsalicylic acid (ASA) and clopidogrel are essential points of secondary stroke prevention and after implantation of neurointerventional and coronary devices [1–3]. Because of their synergistic effect the combination of both drugs induces efficient platelet inhibition and the risk of adverse events like recurrent vessel occlusion and in-stent-stenosis is minimized [4]. Several studies verify lower cardiovascular complication rates in humans treated with dual platelet anticoagulation compared to monotherapies [1, 4–6].

Dual platelet inhibition using ASA and clopidogrel has been shown to effectively reduce complications resulting from intravascularly implanted materials in humans [7–9]. ASA inhibits the formation of thromboxane A_2_, a substance with thrombogenic and vasoconstrictive features, by irreversibly acetylating the thrombocytes’ cyclooxygenase-1 (COX-1) [10, 11]. Clopidogrel is a prodrug, that is activated in the liver and irreversibly binds to the ADP receptor (P2Y_12_ receptor) on the surface of platelets, inhibiting ADP-mediated platelet activation and aggregation [4, 12, 13]. Nevertheless, different studies demonstrate that some patients treated with ASA or clopidogrel show inadequate response to these drugs, so called resistance or low-response [12, 14, 15]. Several mechanisms are discussed but the definitive causes remain unknown. Both, intrinsic and extrinsic factors are of relevance [12, 14, 16]. In clinical studies prevalence rates for ASA resistance have been reported between 5% and 60% [16–20]. This may be attributable to the reliability of tablet intake by patients, but to some extent also to specific tablet formulations (i.e., coated versus uncoated tablets). It is not clear if non-response to intravenously administered ASA exists in humans at all. In 4–51.5% of patients clopidogrel achieved insufficient antiplatelet aggregation [15, 20–23]. To ensure adequate therapeutic response to antiplatelet therapy, platelet function tests are frequently being performed to prevent complications like recurrent vessel occlusion or in-stent-stenosis [24, 25]. Based on these results medication doses can be modified or other platelet inhibitors can be used.

Multiplate® platelet function analysis is a point-of-care method which is based on impedance aggregometry and can be used to measure platelet function in whole blood and thus, examine the response to platelet inhibition. Different tests can be performed using the Multiplate® Analyzer. The tests most commonly used in clinical routine are ASPI test (to assess the effect of the platelet inhibitor acetyl salicylic acid (ASA)), ADP test (adenosine diphosphate test, to assess the effect of the platelet inhibitor clopidogrel), and TRAP test (thrombin receptor activating peptide test, to assess baseline platelet aggregation and to detect the effect of glycoprotein (Gp) IIb/IIIa antagonists). In this way non- or low-responsiveness to ASA and clopidogrel can be detected [26–28].

Dual platelet inhibition is also an important aspect in animal experimental research, especially for testing of (neuro-) interventional devices [29, 30]. In interventional studies the pig is a frequently used species due to its comparable blood vessel sizes to those of humans, which allow to transfer research results to humans [29, 31–34]. As well as in humans, antiplatelet therapy should be monitored in experimental animals to ensure an efficient platelet inhibition.

To our knowledge no studies investigating the applicability of a point-of-care method for analysis of porcine platelet function after platelet inhibition exist. For this reason, the aim of this study was to verify if the Multiplate® Analyzer (Roche, Basel, Switzerland) can be used to examine blood samples of pigs that received ASA and/or clopidogrel for antiplatelet therapy by using the same settings and reagents as for humans. We investigated different settings of drug application (intravenous application, oral application, single drug application, combined drug application) and two different breeds of pigs, which are widespread in research (German Landrace, miniature pig).

## Materials and methods

### Experimental animal groups

All experiments were performed in accordance with the German legislation governing animal studies following the “Guide for the Care and Use of Laboratory Animals” (National Research Council, 8th edition, 2011) and the “Directive 2010/63/EU on the Protection of Animals Used for Scientific Purposes” (EU Official Journal, 2010). The experiments were carried out after receiving approval of the governmental animal care office (Landesamt für Natur, Umwelt und Verbraucherschutz Nordrhein-Westfalen, Recklinghausen, Germany). Institutional guidelines for animal welfare and experimental conduct were followed.

Animals were housed under controlled environmental conditions (20°C±1°C, 12:12 h light/dark cycle). The acclimatization period before starting the experiments was 2 weeks. Apart from fasting directly before the experiments all animals received feed and water ad libitum. Animals were kept in groups of 2 to 4 animals, litter and materials for examination and manipulation were provided.

In summary, we analysed blood samples of 28 female German Landrace (Gerd Heinrichs, Heinsberg-Karken, Deutschland; mean weight of 53.1kg ± 9.8kg, mean age of 5.3 months ± 0.5 month) and 8 female miniature pigs (Ellegaard Göttingen Minipigs A/S, Dalmose, Denmark; mean weight of 38.1kg ± 12.6kg, mean age of 13 months ± 8.4 months) using the Multiplate® Analyzer (Roche, Basel, Switzerland). All animals in the study were part of different experiments (short- and long-term experiments) to investigate new neurointerventional devices, where dual platelet inhibition was required. Platelet inhibition was induced in four different ways: (1) 500mg ASA (Aspirin®, Bayer, Leverkusen, Germany) intravenously, (2) 500mg ASA intravenously and 450mg clopidogrel (Iscover®, Orifarm, Leverkusen, Germany) orally, (3) 250mg ASA orally, (4) 250mg ASA orally and 75mg clopidogrel orally. Oral application of medication occurred during daily feeding, intravenous administration of medication occurred during the experimental setting with anaesthesia.

Standard anaesthesia treatment provides premedication with azaperone (Stresnil 40mg ad. us. vet.; Sanochemia Pharmazeutika AG, Neufeld, Austria), atropin (Atropinsulfat, B.Braun Melsungen AG, Melsungen, Germany) and ketamine (10% Ketavet ad us. vet., Zoetis Deutschland GmbH, Berlin, Germany) followed by intubation. During the experiments the animals were mechanically ventilated with an oxygen air mixture. Anaesthesia was maintained with propofol (Propofol 2% MCT Fresenius; Fresenius Kabi Deutschland GmbH, Bad Homburg, Germany). For analgesia fentanyl (Fentanly-Janssen 0,5 mg, Janssen-Cilag GmbH, Neuss, Germany) was continuously administered. During the experiments the animals were fixed supine, vital functions were monitored. In long-term settings animals were close monitored during recovery from the anaesthetic over a period of 24 hours, pain management was conducted if necessary (NSAI, 4 mg/kg KG Carprofen (Rimadyl ad.us.vet., Zoetis Schweiz GmbH, Zürich, Schweiz)). At the end of the actual experiment animals were sacrificed by intravenous injection of 0.5–1 ml/kg body weight natrium-pentobarbital (Narcoren 16g/100ml;Merial GmbH, Hallbergmoos, Germany).

Standard values for a control group were provided using 36 blood samples of unmedicated pigs (GL n = 28; MP n = 8). These “zero values” were compared to 32 blood samples of animals whose platelet function was inhibited in four different ways: Group 1: Animals (GL n = 10, MP n = 1) were administered intravenously a dose of 500mg ASA (ASA IV). Group 2: Animals (GL n = 5) received an intravenous dose of 500mg ASA in combination with an oral dose of 450mg clopidogrel (ASA IV + clopidogrel PO). Blood samples of group 1 and 2 were always taken seven hours after intravenous drug application to ensure the full drug effect. Group 3: Animals (MP n = 11) were administered orally a dose of 250mg ASA per day for 20 days (ASA PO). Group 4: Animals (MP n = 5) received an oral dose of 250mg ASA in combination with an oral dose of 75mg clopidogrel (ASA PO + clopidogrel PO) per day for at most 84 days. The first blood samples of group 3 and 4 were taken after seven days of drug application, so the full drug effect can be expected. Additional samples were taken at various time points over the whole period of application (20 days for group 3, and 84 days for group 4) to demonstrate the long-term effect of the medication.

### Sample collection and impedance aggregometry

In the context of the experiments the narcotized animals were punctured at the femoral artery and a endovascular introducer sheath was inserted. Blood samples were taken over the sheath only during the experiments with anaesthesia. An amount of 2,7ml blood was filled in hirudin-coated tubes and rested for 30 minutes. Platelet inhibition was measured with a Multiplate® Analyzer (Roche, Basel, Switzerland).

The test was carried out as instructed by the manufacturer. Briefly, in each case 300μl blood was pipetted in three measuring cells, mixed with 300μl of 37°C warm isotonic sodium chloride and incubated for three minutes. 20μl of the relevant reagent (adenosine diphosphate (ADP), arachidonic acid (AA) or thrombin receptor-activating peptide 6 (TRAP-6)) were added. Measurement time of each test (ASPI test, ADP test and TRAP test) was six minutes. Maximum platelet aggregation and aggregation velocity are expressed in arbitrary units (Area under the curve [AUC]).

### Statistical analysis

Continuous variables are presented as mean ± standard deviation if the data follow normal distribution, and as median with interquartile ranges if otherwise. Unpaired Student’s *t*-test or Mann-Whitney *U*-test were used after testing for normal distribution with a Shapiro-Wilk test. P-values with an alpha level ≤0.05 were defined as significant. Statistical analyses were performed using SPSS 23 software (IBM, San Jose, California, USA).

## Results

### Reference values

The mean reference values of all unmedicated pigs (GL+MP; n = 36) were 69.97 ± 16.46 AUC [U], 62.22 ± 19.50 AUC [U] and 13.14 ± 13.66 AUC [U] for the ASPI test, ADP test and TRAP test, respectively.

### Intravenous application of ASA

**Fig 1:**
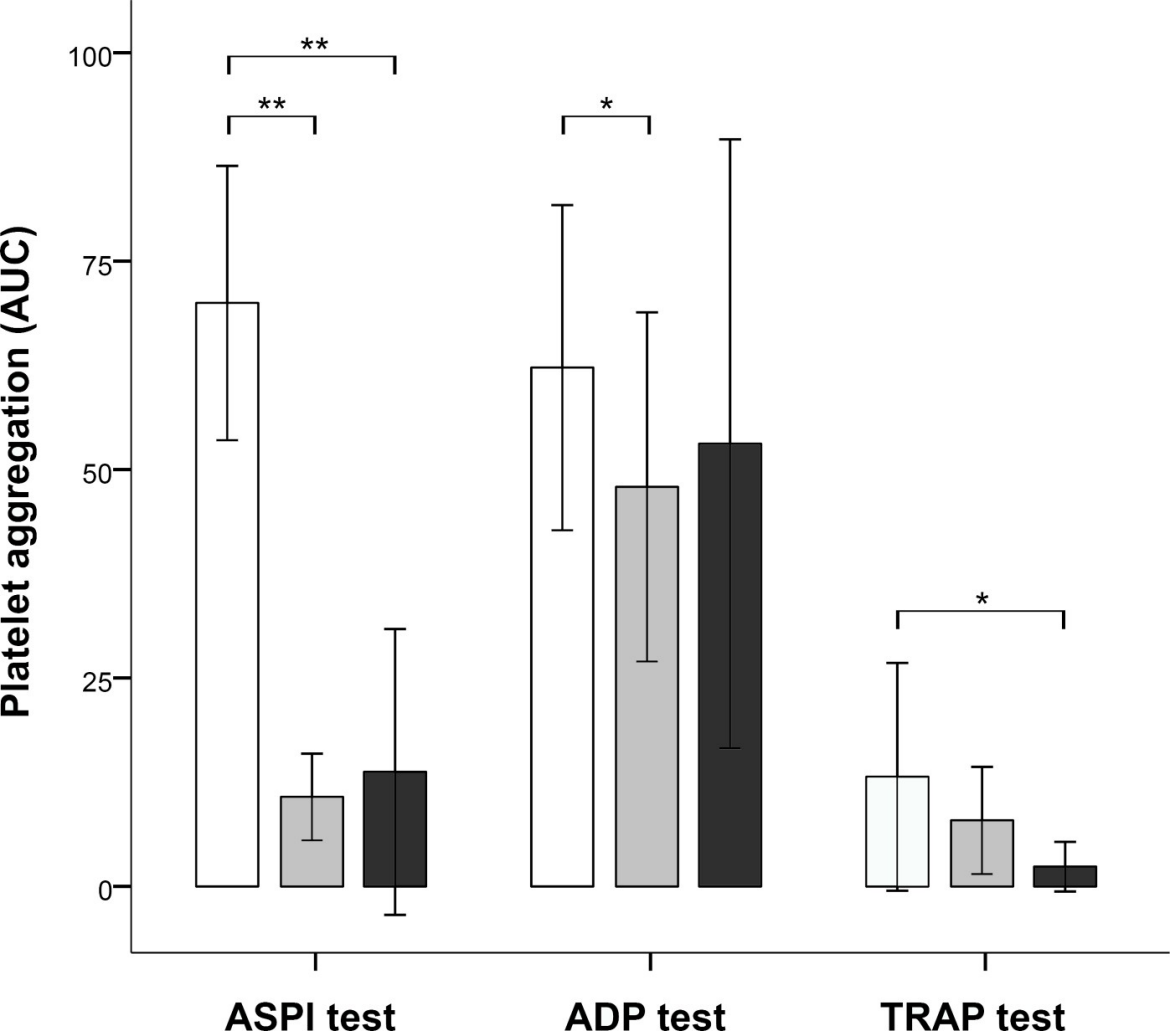
Effects of acetylsalicylic acid (ASA) on platelet function as measured by the Multiplate® Analyzer. Differences between unmedicated pigs (white bars) and pigs following intravenous administration of 500mg ASA (grey bars) reached statistical difference in the ASPI test (p<0.001) and in the ADP test (p = 0.042). After oral administration of 250mg ASA per day for at least 7 days (black bars) differences between unmedicated and medicated pigs reached statistical difference in the ASPI test (p<0.001) and in the TRAP test (p = 0.001).

Intravenous application of ASA (GL+MP; n = 11 / group 1) resulted in significant effects on the ASPI test and the ADP test, but not on the TRAP test. Specifically, we observed significantly decreased mean values of the ASPI test (10.73 ± 5.20 AUC [U], p< 0.001 (*t*-test)) as expected. However, there was also an unexpected significant decrease in the ADP test (47.91 ± 20.95 AUC [U]), p = 0.042 (*t*-test)). Mean values of TRAP test (7.91 ± 6.43 AUC [U], p = 0.443 (*U*-test) [median, 6.00; IQR, 9]) were also decreased, but differences did not reach statistical significance (Fig 1).

### Combined administration of intravenous ASA and clopidogrel

**Fig 2:**
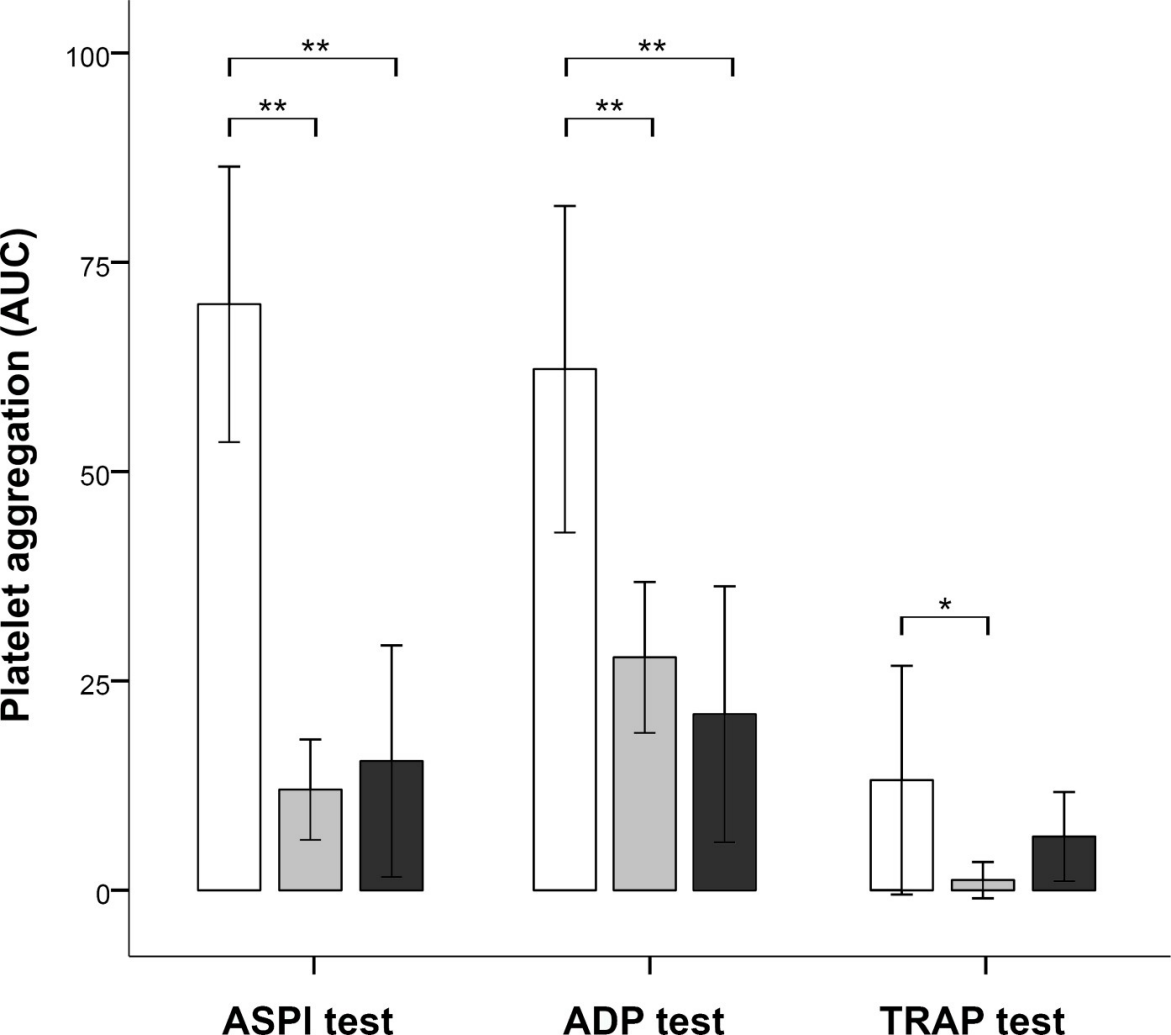
Effects of combined administration of acetylsalicylic acid (ASA) and clopidogrel on platelet function as measured by the Multiplate® Analyzer. Platelet function was determined either 7 hours after combined administration of 500mg ASA intravenously and 450mg clopidogrel orally (grey bars), or 7 days of combined oral administration of 250mg ASA per day and 75mg clopidogrel per day (black bars). Differences between unmedicated pigs (white bars) and pigs receiving short-term medication (grey bars) reached statistical difference in the ASPI test (p<0.001), in the ADP test (p = <0.001), and in the TRAP test (p = 0.005). Differences between unmedicated pigs (white bars) and pigs receiving long-term medication (black bars) reached statistical difference in the ASPI test (p<0.001) and in the ADP test (p = <0.001).

Joined administration of ASA IV and clopidogrel PO (GL; n = 5/group 2) resulted in significant effects on the ASPI test, the ADP test, and the TRAP test. Specifically, we observed significantly decreased mean values for the ASPI test and the ADP test (ASPI: 12.00 ± 6.00 AUC [U], p<0.001 (*t*-test); ADP: 27.80 ± 9.01 AUC [U], p<0.001 (*t*-test)), as expected. However, mean values of the TRAP test were also significantly decreased (1.20 ± 2.17 AUC [U], p = 0.005 (*U*-test) [median, 0.00; IQR, 3]) (Fig 2).

### Oral application of ASA

Oral admission of ASA (MP; n = 11 / group 3) resulted in significant effects on the ASPI test and the TRAP test, but not on the ADP test. Specifically, we observed significantly decreased mean values of the ASPI test (13.73 ± 17.15 AUC [U], p<0.001 (*U*-test) [median, 10.00; IQR, 12]) and, unexpectedly in the TRAP test (2.36 ± 2.98 AUC [U], p = 0.001 (*U*-test) [median, 1.00; IQR, 6]). Mean values of the ADP test were not significantly decreased (53.09 ± 36.52 AUC [U]) (p = 0.442 (*t*-test)), as expected. Both, intravenous and oral administration of ASA induces a comparable decrease in mean values of the ASPI test (Fig 1). As expected, there was no statistical significance between the mean values of the ASPI test of ASA IV and ASA PO (p = 0.792 (*U*-test)).

### Combined administration of oral ASA and clopidogrel

Expectedly, joined oral application of ASA and clopidogrel (MP; n = 5 / group 4) also resulted in significant effects on the ASPI test and the ADP test, but not on the TRAP test. Specifically, we observed significantly decreased mean values for the ASPI test (15.40 ± 13.83 AUC [U], p<0.001 (*t*-test)) and the ADP test (21.00 ± 15.297 AUC [U], p<0.001 (*t*-test)). In this case, mean TRAP test values (6.40 ± 5.32 AUC [U], p = 0.43 (*U*-test) [median, 7.00; IQR, 11]) were also reduced, but differences did not reach statistical significance (Fig 2).

In three cases (GL n = 1; MP n = 2) the values of the ADP test showed no drug effect although the animals received clopidogrel. Considering the conditions of drug application, in two cases drug intake (feeding) could not be guaranteed. However, in one case drug administration was ensured because of intragastral application via a stomach tube. Blood samples of this animal indicated thus a drug inefficacy, probably comparable to a human non-responder. Values of these three animals were not included to the above analysis (Fig 2).

### Comparison between German Landrace and miniature pigs

To analyze differences in platelet inhibition and measurements using the Multiplate® Analyzer between German Landrace and miniature pig, mean values of unmedicated GL (n = 28) and unmedicated MP (n = 8) were compared. There were no significant differences in the ASPI test (p = 0.909; *t*-test) the ADP test (p = 0.12; *t*-test)) and the TRAP test (p = 0.25; *U*-test)) between the two groups. Comparison of mean values for GL (n = 10) and MP (n = 12) after application of ASA (ASA IV + ASA PO) detected no significant differences in the ASPI test (p = 0.668; *U*-test) and the ADP test (p = 0.953; *t*-test) but in the TRAP test (p = 0.010; *t*-test). Furthermore there were no significant differences between mean values of GL (n = 5) and MP (n = 5) after joined application of ASA and clopidogrel (ASA IV + clopidogrel PO and ASA PO + clopdogrel PO) in the ASPI test (p = 0.628; *t*-test), ADP test (p = 0.417; *t*-test) and TRAP test (p = 0.085; *U*-test).

### Adverse effects of platelet inhibition

All animals tolerated the platelet inhibition using ASA and clopidogrel well. Despite drug administration over a longer timeframe (up to 86 days), no gastrointestinal problems or hemorrhagic complications were noted.

## Discussion

Evaluating the effects of dual platelet inhibition is not only important in humans but also in laboratory animals. Presently, there is only sparse experience regarding platelet function analysis in pigs when using a point-of-care method, although antiplatelet therapy is frequently performed in this species.

Normal reference values for platelet function in pigs have been obtained using the ROTEM method [35]. There are small series of platelet function measurements using the Multiplate method in pigs after trauma or hemorrhagic shock [36–39] but data are limited.

Our results suggest that the ASPI test and the ADP test of the Multiplate® Analyzer can be used to analyze porcine platelet function using the common settings and reagents. Expectedly, we measured significantly higher AUC levels of the ASPI test and the ADP test in unmedicated animals as compared to medicated pigs. It is known that arachidonic acid (AA) and adenosine diphosphate (ADP), which are used as reagents in the ASPI test and the ADP test of the Multiplate® Analyzer, are able to induce platelet aggregation in other animal species like horses and dogs [40, 41]. Our results indicate that these reagents can also aggregate porcine platelets and thus can be used to examine platelet function and the response to platelet inhibition in pigs.

It was an unexpected finding that the administration of ASA resulted not only in decreased values of the ASPI test, but also in decreased ADP values. This decrease in ADP values was siginificant after intravenous administration of ASA, but did not reach statistical significance after oral administration of ASA. However, there are recent data indicating that there may be some interference between ASPI and ADP measurements in humans [42]. Further research utilizing larger groups of animals and additional methods for platelet function testing will clarify if this was an accidental finding or indeed point to a difference in thrombocyte function between pigs and humans.

Although none of the animals were medicated with glycoprotein (GP) IIb-IIIa antagonists, all blood samples were examined using the TRAP test. Unexpectedly, measured values of the TRAP test were also decreased after drug application (ASA and / or clopidogrel) compared to values of unmedicated animals. These results suggest that there may be an unknown influence of these drugs to this component of the porcine pathway of platelet inhibition. The effects of GP IIb-IIIa antagonists on platelet function in pigs are unknown, and may be the subject of future research. It has been shown that TRAP-6 is unable to induce platelet aggregation in dogs and sheep [41]. In another study TRAP-14, TRAP-7, TRAP-6, and TRAP-5, promoted full aggregation of platelets in plasma from humans, African Green and Rhesus monkeys, baboons and guinea pigs, but only lead to incomplete response of platelets in rabbits, dogs, pigs, and hamsters [43]. In general, there seem to be large differences in the response of platelets of different species to various stimulating agents [43, 44].

In summary, it seems plausible that the TRAP test of the Multiplate system is not useful in pigs, due to differences of thrombin receptors between species.

In our study blood samples of all animals, which were treated with ASA indicated platelet inhibition as expressed in decreased AUC levels of the ASPI test. Thus, non-response to ASA seems to be rather rare in pigs, if it occurs at all.

In contrast, we found three cases of low- or non-response to clopidogrel, as expressed by weakly decreased, constant or increased AUC levels of the ADP test. Two of these cases were part of a long-term study, where drug ingestion may have been effected by feeding the pills every day over a longer period. For principal reasons it cannot be ensured that the animals ate their pills every day. However, our third case of non-response may be considered a clear result. This animal received clopidogrel over a stomach tube at the beginning of the experiment. Blood samples were taken before and after drug application but AUC levels of the ADP test showed no difference between the samples. Thus low- or non-response to clopidogrel seems to occur in pigs as well, and needs to be considered in interventional studies. However, from a scientific perspective we cannot completely exclude that in these three cases described above the pigs received clopidogrel, the drug was effective, but the ADP test of the Multiplate system is not reliable. Therefore, a considerably larger group of animals is required to define valid cut-off values for clopidogrel “non-response” including confirmatory platelet function tests (e.g., classical platelet aggregometry or VASP analysis). On the other hand, our findings justify such a follow-up study.

Our study showed no differences in the effectiveness of ASA between oral and intravenous application. Furthermore no differences in platelet inhibition between German Landrace and miniature pig were observed.

A limitation of our study is, however, that less miniature pigs were examined and routes of administration of ASA and clopidogrel were not completely identical between German Landrace and miniature pig. This issue results from the fact that the animals in the study were part of different experiments to investigate new neurointerventional devices. Following the 3Rs this limitation seems to be acceptable.

In conclusion, our results indicate that ASA (administered orally or intravenously, respectively) and clopidogrel (administered orally) are able to inhibit platelet aggregation in pigs effectively. The ASPI test and the ADP test of the Multiplate® Analyzer are suitable to examine porcine platelet function, and thus provide the possibility to monitor the effectiveness of antiplatelet therapy in German Landrace and miniature pig.

## Limitations

Since all animals in our study were part of different experiments (short- and long-term experiments) to investigate new neurointerventional devices, the number of animals in different subgroups (e.g., GL versus MP) was not equal, and some subgroups are quite small. For this reason comparisons between subgroups are limited. Specifically, we did not observe differences GL and MP. However, since our study involved only 25 MP pigs, this finding can only be preliminary. In some of our subgroup analyses standard deviations were rather high. Most probably, this is also a consequence of small sample size.

Another limitation of our study is that we did not obtain platelet counts in our pigs. It is a controversial issue if there is some dependence of functional platelet tests on platelet counts. So far, in clinical medicine it is generally accepted that the results of impedance aggregometry do not depend on platelet count if platelet counts are within normal reference range. Only in cases of extremely low platelet counts (< 100 × 10^9^/L platelet range) relevant changes of Multiplate results need to be considered [36, 45, 46]. In our study all pigs were considered healthy by veterinary standards, and platelet counts were expected to be within normal range. However, in some studies a significant correlation between platelet count and aggregation analyzed by the Multiplate was found even for platelet counts within the reference interval [47, 48].

## Supporting information

S1 Table Results of Multiplate® Analyzer measurements. Listed are the results of the Multiplate® Analyzer subtests (ASPI, ADP, TRAP) in German Landrace (GL, Breed = 0) and Minipig (MP, Breed = 1) pigs depending on medication.
(PDF)
