# Supplementary material for: Platelet function testing in pigs using the Multiplate® Analyzer
Source: PLoS One. 2019 Aug 29;14(8):e0222010. doi: 10.1371/journal.pone.0222010 (PMC6715187; doi:10.1371/journal.pone.0222010)
Supplement: S1 Table — Listed are the results of the Multiplate® Analyzer subtests (ASPI, ADP, TRAP) in German Landrace (GL, Breed = 0) and Minipig (MP, Breed = 1) pigs depending on medication. (PDF) [file pone.0222010.s001.pdf]

Unmedicated Animals				
		ASPI	ADP	TRAP
ID 1	Breed	AUC	AUC	AUC
**German Landrace (GL)**				
1	0	**77**	**11**	**10**
2	0	**53**	**50**	**9**
3	0	**72**	**83**	**0**
5	0	**57**	**62**	**4**
7	0	**62**	**43**	**0**
9	0	**82**	**89**	**5**
11	0	**59**	**57**	**6**
13	0	**85**	**70**	**10**
15	0	**42**	**45**	**2**
18	0	**67**	**68**	**40**
22	0	**57**	**46**	**21**
23	0	**72**	**81**	**39**
25	0	**85**	**73**	**20**
27	0	**55**	**52**	**1**
28	0	**88**	**72**	**11**
29	0	**75**	**66**	**7**
30	0	**58**	**56**	**5**
31	0	**31**	**53**	**5**
33	0	**54**	**48**	**0**
35	0	**51**	**58**	**11**
36	0	**67**	**48**	**3**
41	0	**105**	**97**	**16**
43	0	**91**	**84**	**11**
45	0	**67**	**61**	**33**
49	0	**75**	**95**	**43**
51	0	**86**	**71**	**28**
55	0	**92**	**98**	**25**
57	0	**99**	**82**	**54**
**Mean**		**70,14**	**64,96**	**14,96**
**Minipigs (MP)**				
17	1	**49**	**43**	**8**
19	1	**63**	**22**	**5**
20	1	**67**	**52**	**13**
21	1	**76**	**64**	**8**
37	1	**59**	**50**	**4**
38	1	**88**	**75**	**4**
39	1	**79**	**66**	**8**
40	1	**74**	**49**	**4**
**Mean**		**69,38**	**52,63**	**6,75**
**Mean (unmedicated, both breeds)**		**69,97**	**62,22**	**13,14**

Animals receiving ASS (500 mg iv)				
		ASPI	ADP	TRAP
ID 1	Br	AUC	AUC	AUC
4	0	**8**	**40**	**3**
6	0	**16**	**69**	**10**
8	0	**14**	**62**	**6**
10	0	**2**	**73**	**0**
12	0	**5**	**22**	**3**
14	0	**9**	**66**	**12**
16	1	**8**	**18**	**2**
24	0	**17**	**60**	**13**
26	0	**18**	**21**	**22**
32	0	**13**	**60**	**5**
34	0	**8**	**36**	**11**
**Mean**		**10,73**	**47,91**	**7,91**

Animals receiving ASS (250 mg po)				
		ASPI	ADP	TRAP
ID 1	Br	AUC	AUC	AUC
59	1	**6**	**10**	**0**
60	1	**11**	**13**	**1**
63	1	**62**	**47**	**0**
64	1	**18**	**18**	**3**
66	1	**7**	**83**	**2**
69	1	**15**	**75**	**6**
70	1	**17**	**103**	**8**
71	1	**5**	**71**	**0**
72	1	**0**	**47**	**0**
73	1	**0**	**105**	**0**
74	1	**10**	**12**	**6**
**Mean**		**13,73**	**53,09**	**2,36**

Animals receiving ASS (500 mg iv) and Clopidogrel (450 mg po)				
		ASPI	ADP	TRAP
ID 1	Br	AUC	AUC	AUC
44	0	**9**	**20**	**0**
48	0	**17**	**42**	**0**
54	0	**3**	**28**	**1**
56	0	**17**	**29**	**5**
58	0	**14**	**20**	**0**
**Mean**		**12,00**	**27,80**	**1,20**

**Animals receiving ASS (250 mg po) and Clopidogrel (75 mg po)**				
Versuchsnu	Tierart	ASPI	ADP	TRAP
ID 1	Br	AUC	AUC	AUC
61	1	**2**	**9**	**0**
62	1	**5**	**11**	**2**
68	1	**35**	**47**	**7**
75	1	**11**	**17**	**12**
76	1	**24**	**21**	**11**
**Mean**		**15,40**	**21,00**	**6,40**
